# Ground Water Chemistry Changes before Major Earthquakes and Possible Effects on Animals

**DOI:** 10.3390/ijerph8061936

**Published:** 2011-06-01

**Authors:** Rachel A. Grant, Tim Halliday, Werner P. Balderer, Fanny Leuenberger, Michelle Newcomer, Gary Cyr, Friedemann T. Freund

**Affiliations:** 1 Department of Life Sciences, The Open University, Milton Keynes, MK7 6AA, UK; E-Mail: rachelannegrant@gmail.com; 2 21 Farndon Rd, Oxford OX2 6RT, UK; E-Mail: tim.halliday@homecall.co.uk; 3 Swiss Geotechnical Commission, Department of Earth Sciences, ETH Zurich, NO FO 35, 8092 Zurich, Switzerland; E-Mail: werner.balderer@erdw.ethz.ch; 4 Department of Earth Sciences, Geological Institute, ETH Zurich, NO G39.1, 8092 Zurich, Switzerland; E-Mail: fanny.leuenberger@erdw.ethz.ch; 5 Ames Research Center, National Aeronautics and Space Administration (NASA), Earth Science Div. Code SGE, Moffett Field, CA 94035, USA; E-Mail: michelle.e.newcomer@nasa.gov; 6 Department of Physics, San Jose State University, San Jose, CA 95192, USA; E-Mail: garycyr@pacbell.net; 7 Carl Sagan Center, SETI Institute, 189 Bernardo Ave., Mountain View, CA 94043, USA

**Keywords:** earthquakes, positive holes, reactive oxygen species, ROS, hydrogen peroxide, water chemistry, earthquake precursors, animal behavior, amphibians, toads, L’Aquila earthquake

## Abstract

Prior to major earthquakes many changes in the environment have been documented. Though often subtle and fleeting, these changes are noticeable at the land surface, in water, in the air, and in the ionosphere. Key to understanding these diverse pre-earthquake phenomena has been the discovery that, when tectonic stresses build up in the Earth’s crust, highly mobile electronic charge carriers are activated. These charge carriers are defect electrons on the oxygen anion sublattice of silicate minerals, known as positive holes, chemically equivalent to O^−^ in a matrix of O^2−^. They are remarkable inasmuch as they can flow out of the stressed rock volume and spread into the surrounding unstressed rocks. Travelling fast and far the positive holes cause a range of follow-on reactions when they arrive at the Earth’s surface, where they cause air ionization, injecting massive amounts of primarily positive air ions into the lower atmosphere. When they arrive at the rock-water interface, they act as •O radicals, oxidizing water to hydrogen peroxide. Other reactions at the rock-water interface include the oxidation or partial oxidation of dissolved organic compounds, leading to changes of their fluorescence spectra. Some compounds thus formed may be irritants or toxins to certain species of animals. Common toads, *Bufo bufo*, were observed to exhibit a highly unusual behavior prior to a M6.3 earthquake that hit L’Aquila, Italy, on April 06, 2009: a few days before the seismic event the toads suddenly disappeared from their breeding site in a small lake about 75 km from the epicenter and did not return until after the aftershock series. In this paper we discuss potential changes in groundwater chemistry prior to seismic events and their possible effects on animals.

## 1. Introduction

Earthquakes are the most feared among all natural disasters because they seem to strike suddenly, without any forewarning. However, there have been innumerable reports of non-seismic pre-earthquake signals hours, days, and sometimes even weeks before major seismic events. These signals are often fleeting and subtle, seemingly “unreliable”, but occasionally distinct and strong. Many of these pre-earthquake phenomena can reportedly be perceived by animals, eliciting unusual behavior. Reports relating unusual animal behavior to imminent earthquakes date back to antiquity as recounted in Tributsch’s classic book “When the Snakes Awake” [1]. Recent reports of a wide range of physical pre-earthquake phenomena draw on modern science and technology involving ground-based and satellite-based observations. Here is a partial list:

- Low to ultralow frequency electromagnetic emissions from the ground,
- Luminous phenomena, often called earthquake lights, prior to many seismic events,
- Enhanced infrared emission from the epicentral region as seen in satellite images,
- Changes in the atmosphere near the ground and at altitudes up to about 12,000 m,
- Perturbations in the ionosphere 100–600 km above the Earth’s surface,
- Changes in the ocean water and ground or spring water chemistry, *etc.*

Until recently the field of non-seismic pre-earthquake signals was in a general state of confusion. Nobody seemed to be able to identify a physical process, or a sequence of processes, capable of explaining the diversity of the reported pre-earthquake signals or how they may be linked to each other and/or traced back to a physical cause. This lack of understanding has been largely overcome by the discovery of a previously unknown form of electrification when rocks are subjected to mechanical stress [2–4].

## 2. Laboratory and Field Observations

### 2.1. Positive Hole Charge Carriers in Rocks

Rocks are generally thought of as good insulators. However, essentially all rocks in Earth’s crust contain fundamental and seemingly ubiquitous types of defects, which had not been previously recognized. These defects are dormant and electrically inactive. In silicate minerals they consist of peroxy links, e.g., of sites in the structures of common silicate minerals where normal Si/^O^\Si bonds between [SiO_4_] structural units are replaced by Si/^OO^\Si. These are peroxy links and their characteristic feature is that the two oxygens, which form the peroxy bond, have changed their valence state from the usual O^2−^ to the unusual O^−^.

**Scheme 1:**
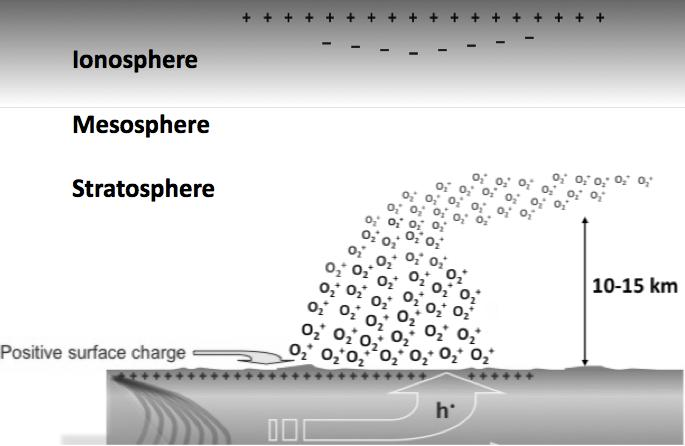
Peroxy bond breakage and generation of a positive hole, h^•^, which become a mobile electronic charge carrier.

In the language of semiconductor physics an O^−^ in a matrix of O^2−^ represents a defect electron or hole, conventionally written as h^•^, also known as positive hole [5]. In the peroxy bond two positive holes are tightly bound, hence electrically inactive. However, when a rock is subjected to mechanical stress, dislocations move through the mineral grains. These moving dislocations cause the peroxy bonds to momentarily break. Scheme 1 depicts how this bond breakage proceeds in two steps, forming at first a short-lived transient state where the two positive holes decouple, followed by an electron transfer from an O^2−^ in the neighborhood. Once transferred, this electron becomes trapped in the broken peroxy bond, while the O^2−^ that has donated the electron changes into an O^−^. It becomes a positive hole h^•^, an electronic charge carrier.

The important point to note is that, in the reaction sequence shown in Scheme 1, two electronic charge carriers are generated: a hole h^•^ and an electron e’. They are both long-lived. As electronic states associated with defect electrons in the O^2−^ sublattice, the h^•^ can move away from the sites, where they were formed. They can propagate not only through the given mineral grain but also jump from grain to grain, spreading from the stressed rock into the unstressed rock [4].

**Figure 1.**
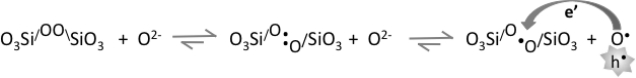
(**a**) The stressed rock volume turns into the source of charge carriers. The positive holes h^•^ flow out into the unstressed rock, creating an electrical potential like in a battery. (**b**) The battery circuit can be closed by running a wire from the pistons (which are in contact with the stressed rock) to the unstressed end of the rock.

This process can be demonstrated in the laboratory by stressing a rock at one end as illustrated in Figure 1a and following the h^•^ as they spread into the unstressed rock. The stressed subvolume, from where the h^•^ flow out, becomes negatively charged. The unstressed rock becomes positively charged. This creates an electrical potential.

In order for the e’ to also flow out, a separate pathway has to be provided. The situation resembles that in an electrochemical battery, where positively charged cations flow out of the anode into and through the electrolyte, while the electrons available in the anode have to wait until they are offered a metal wire to follow suit. In the case of the “rock battery” the positive charges are h^•^. Figure 1b shows that, if a wire is attached to the pistons, which are in electrical contact with the stressed rock volume, and to a Cu electrode at the unstressed end of the rock, the battery circuit is closed allowing a current to flow.

The h^•^ are highly mobile. Once created as illustrated in Scheme 1 they are thought to propagate by way of a phonon-mediated electron transfer mechanism [6]. The experimentally determined maximum speed with which h^•^ charge clouds are able to propagate through solid rock is on the order of 200 m/s, consistent with such a phonon-mediated electron transfer mechanism [4].

Prior to earthquakes tectonic stresses in the Earth’s crust increase, eventually reaching the breaking point of the rocks. During the stress build-up h^•^ charge carriers become activated in increasing numbers. The stressed rock volume thus turns into a battery, from where an h^•^ current can flow out. This is the basic process that provides a comprehensive understanding of a wide range of pre-earthquake signals [2].

The h^•^ streaming out into the surrounding unstressed rocks can travel fast and far, meters in laboratory experiments and probably tens of kilometers in the field. If the battery circuit closes, h^•^ currents can flow continuously, in a quasi-dc mode. Under certain conditions they tend to fluctuate, providing a physically reasonable mechanism to account for the widely reported pre-earthquake ultralow frequency electromagnetic emissions [7–9].

Positive holes flow not only through solid rocks but also through sand and soil. When they reach the Earth surface, the h^•^ can pairwise recombine to form peroxy bonds again. This is an exothermal reaction. It leads to vibrationally highly excited states, which de-excite by emitting infrared photons [10]. The infrared emission thus created may be responsible for the widely reported pre-earthquake “thermal anomalies” captured in night-time infrared satellite images of the areas around future epicenters [11–13]. In addition the arrival of h^•^ charge carriers at the surface leads to the buildup of surface/subsurface charges, which generate microscopic electric fields, strong enough to ionize the air and inject massive amounts of airborne ions at the ground-to-air interface [14]. The surface charges can cause corona discharges accompanied by visible light [4,15] and by noise in the radiofrequency range, which can interfere with telecommunications [16].

Air laden with positive ions leads to condensation of water droplets, causing haze and clouds. Expanding upward, the ionized air will carry along the Earth’s ground potential to stratospheric heights. The resulting changes in the vertical electric field are expected to affect the ionosphere and may be the cause of ionospheric perturbations [17–19] and changes in the transmission of radiowaves [20–22], which reportedly precede major earthquakes by a few days.

Positive airborne ions cause the blood serotonin level to increase, both in humans and animals [23]. Thus, changes in the serotonin levels as result of a pre-earthquake injection of positive airborne ions into the Earth’s near-surface atmosphere may be instrumental in causing the widely reported anomalous behavior of land animals prior to major earthquakes [14,24,25], in causing an increase in migraine incidences in humans [26] and the general response of humans to air masses laden with positive ions [27,28].

In this paper we consider what happens when h^•^ charge carriers flow into water, and what type of chemical reactions occur at the rock-water interface that change the water chemistry and may affect animal behavior.

### 2.2. Oxidation of Water to Hydrogen Peroxide

**Figure 2.**
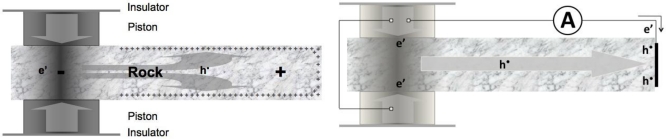
Schematic representation of the flow of a positive hole, e.g., a defect electron on the O^2−^ sublattice through a sequence of electron transfer steps from left to right. (**a**) at the rock-water interface the positive hole appears as an O^−^. (**b**) an O^−^ is a highly oxidizing •O radical that can subtract an H atom from H_2_O, thereby generating an •OH radical in solution.

Figures 2a/b depict that, while an h^•^ moves in one direction, electrons e’ hop in the opposite direction through a succession of O^2−^, which turn momentarily into O^−^. Figures 2a/b illustrate five electron transfers from right to left, while the h^•^ moves five positions from left to right.

Physically an h^•^ is an electronic charge carrier but chemically it is an O^−^, an open shell configuration with 7 electrons in its outer shell. Such an O^−^ acts as a highly oxidizing •O radical, in biochemistry generically known as “reactive oxygen species”, ROS.

Upon arriving at the rock-water interface, as depicted in Figure 2a, the h^•^ acts as O^−^, capable of oxidizing an H_2_O molecule. As the O^−^ subtracts an H from H_2_O, it changes into an OH^−^, which remains imbedded in the rock surface. At the same time the H_2_O molecule turns into an •OH radical as depicted by Figure 2b. Two •OH radicals combine to form hydrogen peroxide:

(1a) $${\text{O}}_{\text{surface}}^{-}+{\text{H}}_{2}{\text{O}}_{\text{solution}}Ȳ{\text{OH}}_{\text{surface}}^{-}+•{\text{OH}}_{\text{solution}}$$

(1b) $$•{\text{OH}}_{\text{solution}}+•{\text{OH}}_{\text{solution}}Ȳ{\text{H}}_{2}{\text{O}}_{2(\text{solution})}$$

Experimental data [29] confirm that this reaction is stoichiometric with one H_2_O_2_ forming for every two h^•^, which cross the rock-water interface.

### 2.3. Open Circuit *versus* Closed Circuit

As h^•^ flow from one point to another they create a potential difference. The closest analog is the outflow of cations from the anode of an electrochemical battery creating a “battery voltage”. When the battery circuit is open, as depicted in Figure 1a, the potential counteracts the charge outflow and soon brings it to a halt. Hence, the number of charge carriers that can flow out of the anodic volume is limited. In order to establish a continuous outflow current, it is necessary to close the circuit, as depicted in Figure 1b, allowing electrons to also flow out. This analogy also applies to the rock battery.

**Figure 3.**
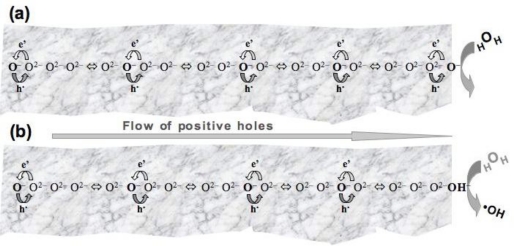
(**a**) Basic laboratory set-up to demonstrate that stress-activated h^•^ currents can flow into water and close the battery circuit. (**b**) Demonstration of a rock battery where the circuit closure is achieved through the electrolytical conductivity of water.

Of course, in the Earth’s crust, there are no metal wires connecting different volumes of rock that are subjected to different levels of tectonic stress. However, Nature provides other alternatives to close the “rock battery” circuit [2]. One possibility is to establish an electrolytically conductive pathway. Figure 3a illustrates a laboratory set-up where the h^•^ are allowed to flow from the stressed rock volume into a water reservoir attached to the unstressed end of the rock sample. As the h^•^ enter the water, they react to form H_2_O_2_ at the rock-water interface as described by Equations 1a,b, but the current continues through the bulk of the water, presumably by way of H_3_O^+^ [29]. When the H_3_O^+^ reach the Cu electrode, they are expected to react with the electrons that have traveled through the metal wire from the stressed rock volume to the Cu electrode:

(2) $$2\;\text{e}’+2\;{\text{H}}_{3}{\text{O}}^{+}Ȳ2\;{\text{H}}_{2}\text{O}+{\text{H}}_{2}$$

Equation 2 predicts that closure of the rock battery circuit is possible by electrolyzing water to H_2_O_2_ plus H_2_ [29]. If this is true, we can get rid of the metal wire plus Cu electrode altogether and close the rock battery circuit by connecting the water reservoir at the unstressed end of the rock with a water reservoir that is in contact with the stressed rock volume.

Figure 3b illustrates the experimental set-up used to demonstrate closure of the rock battery circuit through an entirely electrolytical pathway. Formation of H_2_O_2_ in the water reservoir at the unstressed end of the rock was used to verify that the circuit closure had indeed taken place [29].

### 2.4. Electrocorrosion of Rocks

According to Equation 1a the surface of a rock, through which h^•^ charge carriers flow into water, must become hydroxylated. This is equivalent to saying that the rock undergoes accelerated weathering or some form of electro-corrosion. Expected consequences of such a process are that, while h^•^ flow into the water, the pH values become more acidic and more cations will be released from the rock surface than during normal rock-water interactions.

There have been reports in the literature that, prior to earthquakes, the concentration of dissolved cations in spring and ground water often increases, for instance at ground water measuring stations along the North Anatolian Fault in Turkey [30]. Moderate seismic activity in the otherwise stable Northwestern part of Spain was preceded by several weeks by distinct changes in the water chemistry including increases in electrical conductivity due to dissolved cations and anions [31]. Likewise the pH values in deep boreholes in Kamchatka, Russia, reportedly indicated increased acidity of deep well waters prior to major earthquakes in the region [13,32].

**Figure 4.**
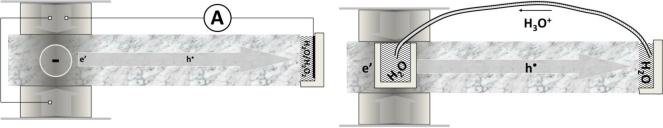
Experimental set-up to demonstrate the accelerated dissolution (electrocorrosion) of the rock surface while h^•^ charge carriers flow into the water reservoir for which circuit closure has been established.

The electrocorrosion of rocks can be demonstrated in the laboratory. Figure 4 depicts a slab of gabbro, 30 × 60 × 9 cm^3^, with identical water reservoirs attached to both ends. Both reservoirs contain a 20 × 6 cm^2^ Cu electrode separated from the rock surface by 1 cm of water. Applying a constant load of about 10 tons to the center of the gabbro slab through a pair of stainless steel pistons, 5 × 5 × 30 cm^3^, activates h^•^ charge carriers and causes them to flow out as indicated by gray arrows. Figure 4 shows circuit closure for the water reservoir on the right but not for the one on the left.

**Figure 5.**
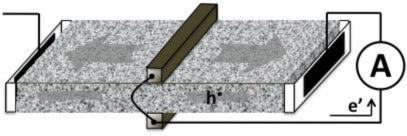
(**a/b**) Excess cation concentrations measured in the water reservoir attached to the slab of gabbro into which h^•^ were allowed to flow continuously over a period of 10 weeks.

Figures 5a/b shows the differences in the concentrations of dissolved K^+^, Ca^2+^ and Mg^2+^ measured between the two water baths as a function of time, while the load on the rock was kept constant. Starting in the second week the dissolved cation contents increased in the water bath for which circuit closure had been established, indicating accelerated dissolution of the rock surface.

### 2.5. Other Oxidation Reactions at the Rock-Water Interface

If h^•^ charge carriers have the capability to oxidize H_2_O to H_2_O_2_ at the rock-water interface, there are reasons to expect that they will also have the capability to cause other oxidation reactions. Of special interest are oxidation reactions involving organic compounds of biogenic origin dissolved in spring and ground water.

Groundwater samples were collected in July 1999 within the zone of influence of the North Anatolian Fault Zone (NAF). After the highly destructive M7.6 Izmit earthquake of August 17, 1999 further samples were taken in order to study possible effects of this earthquake on the isotopic and chemical compositions of thermal and mineral waters in the surrounding areas [33]. Significant changes were also observed in the intensities of the fluorescence spectra of thermal and mineral waters collected prior to the Izmit earthquake in the nearby areas of Kuzuluk, Bursa, and Yalova/Gemlik [34].

**Figure 6.**
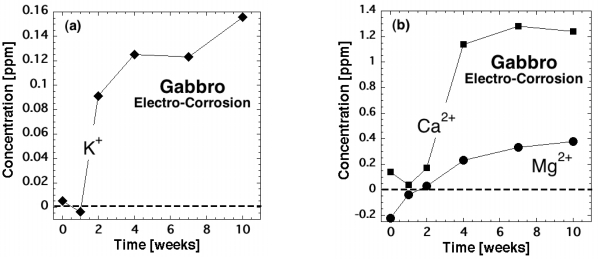
Fluorescence spectra of water samples of the Cekirge (Bursa) Vakif Bahçe Spring, Turkey (sampling 1999 to 2003).

Figure 6 shows fluorescence spectra of water samples from a location near Bursa, Turkey. Sample #16, collected July 10, 1999, 7 weeks before the earthquake, exhibits a slightly elevated 340 nm intensity.

In sample #33, collected October 6, 1999, during intense aftershock activity about 7 weeks after the Izmit earthquake, the 340 nm peak intensity is significantly higher. One year later, in sample #47, the fluorescence intensity at 340 nm had decreased, and remained low over the course of the next 3 years, though sample #90, collected Aug. 2003, exhibited a higher fluorescence intensity around 280 nm.

**Figure 7.**
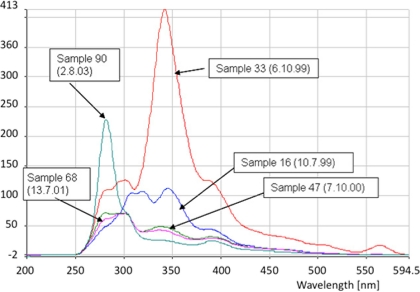
Water samples collected on the shores of Lago di Garda, Italy, prior to and after a modest M5.3 earthquake, 25 km focal depth, 8 km from Salò.

Another example comes from the Lago di Garda, Italy, where an M5.3 earthquake occurred on Nov. 24, 2004, at a focal depth of 25 km, 8 km from the village of Salò [35]. Figure 7 shows the fluorescence spectrum #1 of a reference water sample from the Tavina Mineral Spring at Salò and the spectra of several water samples collected about 17 days before the earthquake, #2, #3, and #4 from three open outlets of the Tavina spring, #5 from a fountain on the road to Gardone, and #6 from an adjacent creek.

**Figure 8.**
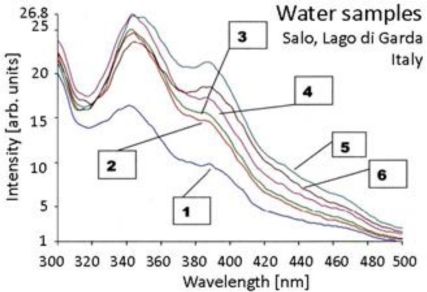
Terephthalate assay: partial oxidation of the aromatic ring through the reaction with an •O radical, leading to a diagnostic change in the fluorescence spectrum [36].

The observed changes in the florescence indicate that existing dissolved organic compounds spectra in the groundwater and spring water become partially oxidized. A well-known example of this type of reaction is the oxidation of terephthalate, an aromatic dicarboxylic acid, used as an assay for ROS, reactive oxygen species [36]. As Figure 8 shows the reaction with an •O radical leads to the addition of an O to the aromatic ring, changing the fluorescence of the molecule.

Which specific organic compounds were affected in the groundwater and spring water samples depicted in Figures 5 and 6 is not known at this time. However, the observations points to oxidative reactions by h^•^ charge carriers that become activated in the stressed rocks within the hypocentral region of an impending earthquake or during the aftershock series. These h^•^ charge carriers will flow down stress gradients and become available over a wide area, tens of kilometers in diameter, surrounding the sites of maximum seismic activity. While propagating through the near-surface soil, the h^•^ will enter the water table from below. They are expected to cause a wide range of oxidation reactions. If these reactions involve organic matter dissolved in the groundwater or adsorbed to soil particles, the products may include partially oxidized ketones and carboxylic acids, which would remain in solution, all the way to carbon monoxide, CO, which can be emitted as a gas from the soil.

Massive amounts of CO were released from the ground 4–6 days before the M7.6 Gujarat earthquake of January 26, 2001 in Northwest India, enough to be detected in spectrally resolved daytime and night-time images of the MOPITT sensor onboard the NASA TERRA satellite [37]. Since the CO concentration was highest at ground level, reaching an average of 0.25 ppmv (volume parts per million) between ground and about 6000 m altitude, the CO was clearly associated with an emanation from the soil.

### 2.6. Unusual Animal Behavior before Earthquakes

Unusual animal behavior before major earthquakes has been reported through the ages [1,38]. Apparently, during the build-up of stresses deep in the Earth crust to dangerously high levels, many animals are able to perceive cues from the environment, which cause them to react abnormally. Animals both on land and in water are reportedly affected. Evidence for unusual animal behavior has been widely reported in the past as a warning sign of impending major earthquake activity, though such reports have widely been called anecdotal [24,39]. Since earthquakes are rare events, reports of unusual animal behavior are—by their very nature—in almost all cases anecdotal [24,39].

Attempts to validate the unusual animal behavior in laboratory experiments have been largely inconclusive [25,40]. However, as pointed out in a recent review of the 1975 Haicheng earthquake in China [41]: “Among the animals, the most difficult to ignore are the snakes coming out hibernation dens when the average temperature was much below freezing. There were nearly 100 snake sightings within one month prior to the earthquake [42]… such suicidal behavior is extremely difficult to explain”.

A recent example of unusual animal behavior has been given by Grant and Halliday, who reported that the activity of common toads at a breeding site in a small lake about 75 km north of L’Aquila, Italy, declined dramatically five days before the M6.3 L’Aquila earthquake on April 6, 2009 [43]. The apparent abandonment of the breeding site and interruption of spawning is highly unusual in these amphibians, which exhibit an explosive breeding behavior. Once the breeding activity has commenced, the toads normally do not leave the site for 3–7 weeks until spawning is completed [44]. Although the breeding site was some distance from the epicentre, it has been shown that there was a major extension of earthquake related phenomena to the north of the epicentre, including earthquake lights and electric anomalies [45]. A similar effect seemed to have occurred before the great 1873 earthquake in central Italy as reported by Serpieri, who observed unusual snake behavior in his laboratory upon application of electric currents [46].

**Figure 9.**
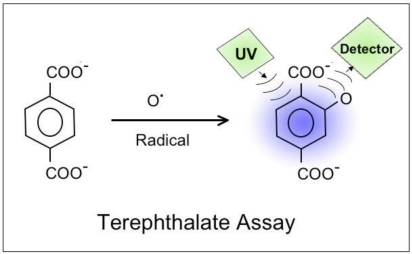
(**top**) Number of male toads observed at the lake site; (**bottom**) Seismic activity associated with the M6.3 L’Aquila earthquake of April 06, 2009 including foreshock, main shock and aftershock activity.

Figure 9(top) shows for the period of March 26 to April 17, 2009 the number of male toads observed per day. Figure 9(bottom) shows the L’Aquila seismic sequence, which includes the M6.3 main shock at 3:32 am on April 06 [43]. During 5 days leading up to the main shock the number of male toads observed dropped to near-zero. Their number recovered slightly after the main shock and dropped again, though not to near-zero, prior to the major aftershock of April 13.

**Figure 10.**
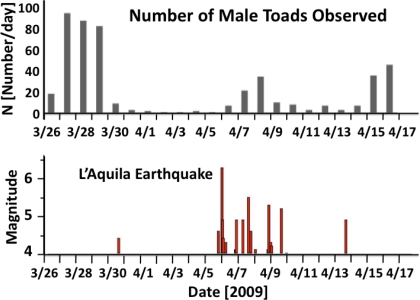
Rainfall records for the lake site and at a weather station at a distance of 15 km.

Figure 10 shows the rainfall recorded both locally at the site and at a weather station about 15 km away from the lake. Since the period of highest rainfall between March 30 and April 04 coincides with the apparent disappearance of the toads from their breeding site, lack of precipitation can definitely not be the cause of the unusual toad behavior.

The rainfall measurements at the breeding site and contemporary field observations indicate that the ground was highly saturated in the week prior to the earthquake. Under normal circumstances such conditions would be advantageous to toads, which are prone to desiccation because of their permeable skins and always prefer humid or damp environments. The disappearance of the toads from the breeding site is all the more unusual, as they would be expected to be more active during rainy periods [47].

The time period during which the toads left the lake coincided with other suspected pre-earthquake indicators such as disturbances in the ionosphere. These disturbances consist of changes in the transmission characteristics of radiowaves along direct great circle paths between two stations. The changes arise between sunset and sunrise, e.g., during the dark hours, when the ionosphere above the region of interest, in this case central Italy, moves out of and back into the influence of the ionizing solar radiation, respectively. Processes that occur at the Earth surface before major earthquakes have an influence on the concentration profile of free electrons in the ionospheric plasma [48,49]. Electrons contribute primarily to the mirror-like reflection of radiowaves, which allows them to be transmitted over long distances. Therefore a change in the vertical distribution of electrons in the ionosphere causes a distinct shift in the so-called terminator times, e.g., in the transmission characteristics around sunset and sunrise [50,51].

**Figure 11.**
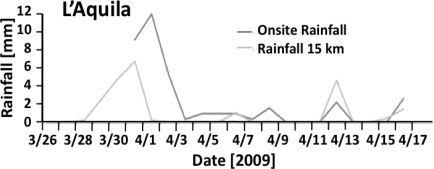
Evidence for ionospheric disturbances along the radio transmission paths between two emitter stations in Italy, ICV in Sardinia and ITS in Sicily, and a receiver station MOS in Moscow, Russia. The disturbances occur between sunset and sunrise and are due to changes in the times at which the radio signals can be received at a certain intensity in Moscow [20].

Figure 11 shows an example of such ionospheric disturbances as they expressed themselves in the difference between the amplitudes (dAmplitudes) of radio signals emitted from two stations in Italy, ICV at 20.27 kHz in Sardinia and ITS at 45 kHz in Sicily, and received at the MOS station in Moscow, Russia. On their path to MOS in Moscow, Russia, the ICV and ITS signals pass over the ionospheric region that lies within the sphere of influence of the L’Aquila earthquake [20]. By referencing the signals along the path ICV-MOS and ITS-MOS to signals received in Moscow from radio stations in England and Iceland, it can be shown that the ionosphere above central Italy was perturbed several days before the L’Aquila event, giving rise to anomalous negative intensities (dAmplitude) as marked by dotted circles.

Unusual pre-earthquake conditions in the ionosphere above the region of L’Aquila as presented here [20] have been confirmed by other authors [52]. Electromagnetic signals ranging from ultralow to kHz and MHz frequencies have been recorded around the same time [53–55]. In addition there have been a number of reports of flashes of visible light and of “cold flames” breaking out of the ground at distances up to 50 km from the L’Aquila epicenter [45]. Such visible light phenomena have an electric origin and are known as “earthquake lights” [56,57].

## 3. Discussion

Unusual animal behavior before major earthquakes has been reported through the ages [1]. There are reports of anomalous behavior relating to fish, amphibians, and reptiles. Deep-sea fish rising to the surface have been observed on numerous occasions, as have fish jumping out of the water [58,59]. The deep sea species *Trachipterus ishikawae*, a ribbon fish, and *Regalecus russelii,* the oarfish, are commonly seen before earthquakes [60].

Semi-aquatic animals have the capacity to leave the water, whereas fully aquatic species do not have this option. Crabs have been seen leaving the water in large numbers prior to earthquakes and unusual movements of frogs and toads have been noted as a possible pre-earthquake indicator [43,58,59], including mass migrations of toads prior to the M8.0 May 12, 2008 Sichuan Earthquake [61]. Similarly, prior to the M7.3 February 04, 1975 Haicheng earthquake in northern China, hundreds of snakes were observed coming out of hibernation in subzero temperatures [41], a behavior that is highly unusual and would generally be considered maladaptive in this ectothermic group of animals.

However, much unusual pre-seismic animal behavior probably goes unreported in the scientific literature for several reasons: (i) The topic has become unfashionable among biologists as *post hoc* evidence is largely viewed as selective recognition [60], (ii) isolated incidents of unusual behavior are difficult to link conclusively to earthquakes, and (iii) the unpredictable nature of earthquakes means that biologists are rarely at the scene to record at the time of seismic events. An exception to this last rule is provided by the fortuitous observation of unusual behavior of toads in the lake near L’Aquila, Italy, prior to the M6.3 April 6, 2008 earthquake, which was part of a systematic 4-year study of toad breeding behavior at this very location [43].

Earth is a highly dynamic planet. Plate tectonic movements continuously cause a waxing and waning of stresses in the Earth’s crust [62]. The strain is usually accommodated by the slow deformation of rocks and/or soft sliding along faults. Occasionally, however, stresses in the crust build up to dangerously high levels leading to catastrophic failure of rocks and to earthquakes.

What had not been previously recognized is the fact that, during the build-up of stresses, electronic charge carriers are activated through the breakage of pre-existing, yet dormant peroxy defects in the matrix of rock-forming minerals [2]. As illustrated in Scheme 1 this process leads to positive hole charge carriers, h^•^, which have the unusual property that they are able to flow out of the stressed rock volume and to spread into the surrounding less stressed or unstressed rocks. The h^•^ can travel fast and far, meters in laboratory experiments, presumably kilometers to tens of kilometers in the field. The h^•^ are not only highly mobile, but also have a dual nature. On one hand they are charge carriers, whose propagation through the Earth crust constitutes an electric current. On the other hand, because the h^•^ represent an O^−^ state in a matrix of O^2−^, they are chemically equivalent to •O radicals. As such they are highly reactive and highly oxidizing, capable of executing a variety of reactions, when they arrive at some common discontinuities in the environment such as the rock-air interface and the rock-water interface.

At the rock-air interface the h^•^ build up surface/subsurface charges, which have long been suspected to become strong enough—at sufficiently high number densities—to “rip off” an electron from air molecules [63] and thus inject positive airborne ions into the atmosphere [14].

At the rock-water interface the h^•^ have been shown to be able to extract an H from H_2_O molecules, thereby forming •OH radicals, which in turn form H_2_O_2_ as depicted in Figures 2a/b [29].

These two basic processes, plus the fact that h^•^ flowing through the Earth crust constitute an electric current, allow us to look at the diversity of reported pre-earthquake phenomena in a new way and to see correlations between seemingly disjointed observations such as the anomalous behavior of toads at the peak of their breeding season in small lake near L’Aquila, Italy, and ionospheric disturbances above central Italy during the days before a M6.3 earthquake which inflicted heavy damage to the town of L’Aquila and loss of life.

We thus begin to understand why, during the propagation of h^•^ charge carriers through the rocks, one should expect to see ultralow frequency electromagnetic emissions, such as reported in the literature [9,64–66]. We also begin to understand why, upon arrival of large numbers of h^•^ at the rock-air interface over a region of tens of kilometers across, possibly even larger, the air becomes ionized and large amounts of airborne ions, primarily positive ions, are injected at the rock-air interface as experimentally demonstrated [14].

**Figure 12.**
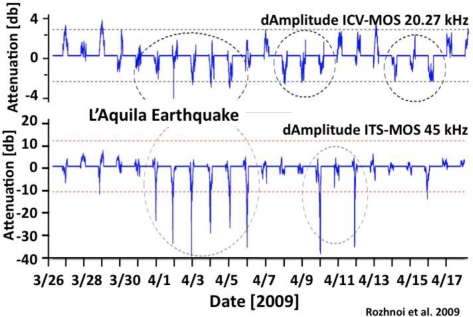
Concept drawing of the effect that an ion-laden air bubble, forming at ground level and rising through the atmosphere, will have on the ionosphere.

Figure 12 depicts schematically such a situation where tectonic stresses build up inside the Earth crust (lower left) leading to the activation of an ever increasing number of h^•^ charge carriers. The h^•^ flow out of the stressed rock volume. They are believed to be able to spread tens of kilometers down stress gradients and to the Earth surface.

As the number density of h^•^ charge carriers at the surface increases, air molecules become field-ionized, probably O_2_, generating large amounts of positive airborne ions at the rock-air interface. Prior to earthquakes episodes of air ionization have been observed [9]. Air laden with positive ions will expand upward, probably to stratospheric heights, dragging along the Earth’s ground potential. The potential difference between the ground and the ionosphere is on the order of 250,000 V [67]. Therefore, as the ion-laden air bubble rises, it will alter the vertical electric field and cause the ionospheric plasma to respond accordingly. As indicated in Figure 12, electrons in the ionospheric plasma are predicted to be pulled downward. This will lead to an increase of the Total Electron Content (TEC) at the lower edge of the ionosphere [68–70] and to a change in the radio wave transmission characteristics as mentioned above.

If waves of h^•^ charge carriers arrive at the Earth surface over a wide area surrounding a future epicenter and if the number of h^•^ becomes so large in the days before a major earthquake as to ionize the air [14] and cause an ionospheric response, then we can expect that the same waves of h^•^ charge carriers will enter into the water table and into bodies of water in the affected region. Crossing into the water the h^•^ will oxidize H_2_O to H_2_O_2_ as experimentally demonstrated [29], will oxidize or partially oxidize organic compounds dissolved in the water as suggested by the reported changes in the fluorescence spectra of ground and spring water samples from seismically active areas [34], and will acidify the water. Some partial oxidation products may have an O atom attached to an aromatic ring as in the case of terephthalate mentioned in the context of Figure 8 or may consist of ketones, carboxylic acids or CO. At the same time the pH is expected to drop as the water becomes acidified.

We will now discuss the potential effects of some of these changes on biological systems. Under non-extreme conditions, cells produce a variety of enzymes and non-enzyme antioxidants as a defense against oxidative stress, and there is a balance between oxidant and antioxidant processes. However, once the balance is upset, severe oxidative stress can lead to cell death by apoptosis and tissue necrosis [71,72]. There are many studies, which demonstrate the time- and dose-dependent cytotoxic effects of H_2_O_2_ on lipids in the cell membrane [73], on cell proteins [74], and on DNA [75]. Hydroxyl radicals (•OH) are highly reactive and very damaging, due to their capacity to react with any biological molecule, causing free radical chain reactions, the oxidation of membrane lipids and denaturing of proteins, thus inactivating important enzymes required for biological functioning [75]. Low pH has also been shown to detrimentally affect cells and whole animals for example, by reducing protein synthesis, which negatively affects growth and reproduction [76,77]. Some of the oxidation products of dissolved organic matter are neurotoxins [78] and furthermore carbon monoxide can cause death by binding preferentially to hemoglobin instead of oxygen, forming carboxyhemaglobin, so that oxygen cannot be delivered to the tissues and organs of the body. High concentrations of cations may affect the water and sodium balance of aquatic animals.

Amphibians are particularly sensitive to changes in water chemistry as their skins are permeable to electrolytes [79]. Several anions and cations have been found to adversely affect the survival of amphibians at different life history stages [80]. The presence of certain metal ions increases the damaging effects of H_2_O_2_ [81] and exposure to H_2_O_2_ can lead to limb abnormalities in larval amphibians [82]. If CO is produced by the oxidation of organic matter in the soil or water column, it can lead to neuropsychological impairment, even at low levels [78]. Low pH in breeding ponds has been shown to cause both lethal and sublethal effects in a range of amphibian species [83] by inhibiting active uptake of Na^+^ and increasing the loss of passive ions through amphibian skin [79]. Sodium ions (Na^+^) are actively transported across amphibian skin from the environment (Cl^−^ follows passively) and the presence of high concentrations of H^+^ K^+^, Mg^2+^ and Ca^2+^ interferes with this process [84]. The inability of amphibians to maintain their sodium balance can quickly lead to death.

The combined evidence provided by the toad observation at the lake near L’Aquila and the ionospheric disturbance data as derived from radiosounding suggests that the toads were able to perceive in their environment some pre-seismic cues, which warned them of the impending earthquake. The toads may have responded to pre-seismic signals as a simple avoidance reaction to an adverse stimulus or as an evolved adaptation [25]. A possible reason for the toads’ apparent movement to higher ground [43] might be found in an evolutionarily imprinted response to the danger of landslides and flooding. However, both of these explanations lack credibility, in particular the flooding argument because common toads are semi-aquatic during the breeding season and would be unlikely to leave flooded lowlying land around the lake.

The analysis presented here approaches the coincidence of unusual toad behavior and ionospheric disturbances from a different perspective. Both phenomena are driven by a physical process in the Earth crust, in the future hypocentral volume, by the activation of h^•^ charge carriers during the rapid increase in tectonic stress prior to the seismic event. As h^•^ charge carriers spread out in ever larger numbers from the most severely stressed rock volume deep below, they cause different secondary processes at the land surface and at the rock-water interface. The process at the land surface consists of massive air ionization, which has a measurable effect on the ionosphere. The process at the rock-water interface consists of changes in the groundwater and presumably lake water chemistry, which seems to have provided the toads with an impetus to leave and seek refuge on higher ground. Thus, the toads did not react in response to the ionospheric disturbances *per se* but to cues that resulted from chemical reactions at the ground-water interface, which were driven by the arrival of stress-activated h^•^ charge carriers from deep below. Their flight from adverse or toxic environmental conditions provides a more parsimonious explanation than the suggestion that animals might possess an evolved response to specific pre-earthquake conditions [25].

There is little doubt that anomalous animal behavior does occur prior to major earthquakes. Given the variety of physical and chemical processes documented to occur over the large earthquake preparation zone [85], it would in fact be surprising if animals were not affected. In this paper, we have suggested a possible common mechanism for diverse physical pre-earthquake processes and incidents of anomalous animal behavior. How this information may be used for forecasting earthquake risk will be the subject of future research.

## References

[1] Tributsch H (1984). When the Snakes Awake: Animals and Earthquake Prediction.

[2] Freund FT (2010). Toward a unified solid state theory for pre-earthquake signals. Acta Geophys.

[3] Freund FT, Takeuchi A, Lau BW (2006). Electric currents streaming out of stressed igneous rocks—A step towards understanding pre-earthquake low frequency EM emissions. Phys. Chem. Earth.

[4] Freund F (2002). Charge generation and propagation in rocks. J. Geodyn.

[5] Griscom DL (1990). Electron spin resonance. Glass Sci. Technol.

[6] Shluger AL, Heifets EN, Gale JD, Catlow CRA (1992). Theoretical simulation of localized holes in MgO. J. Phys. Condens. Matter.

[7] Kushwah V, Singh B, Hayakawa M (2005). Ultra low frequency (ULF) magnetic field anomalies observed at Agra and their relation to moderate seismic activity in Indian region. J. Atmos. Sol.-Terr. Phys.

[8] Fraser-Smith AC (2008). Ultralow-frequency magnetic fields preceding large earthquakes. EOS.

[9] Bleier T, Dunson C, Maniscalco M, Bryant N, Bambery R, Freund FT (2009). Investigation of ULF magnetic pulsations, air conductivity changes, and infra red signatures associated with the 30 October 2007 Alum Rock M5.4 earthquake. Nat. Hazards Earth Syst. Sci.

[10] Freund FT, Takeuchi A, Lau BWS, Al-Manaseer A, Fu CC, Bryant NA, Ouzounov D (2007). Stimulated thermal IR emission from rocks: Assessing a stress indicator. eEarth.

[11] Tramutoli V, Cuomob V, Filizzolab C, Pergolab N, Pietrapertosa C (2005). Assessing the potential of thermal infrared satellite surveys for monitoring seismically active areas: The case of Kocaeli (Izmit) earthquake, August 17, 1999. Remote Sens. Environ.

[12] Ouzounov D, Freund FT (2004). Mid-infrared emission prior to strong earthquakes analyzed by remote sensing data. Adv. Space Res.

[13] Tronin AA, Molchanov OA, Biagi PF (2004). Thermal anomalies and well observations in Kamchatka. Int. J. Remote Sens.

[14] Freund FT, Kulahci I, Cyr G, Ling J, Winnick M, Tregloan-Reed J, Freund MM (2009). Air ionization at rock surface and pre-earthquake signals. J. Atmos. Sol.-Terr. Phys.

[15] Araiza-Quijano MR, Hernández-del-Valle G (1996). Some observations of atmospheric luminosity as a possible earthquake precursor. Geofis. Int.

[16] Kolvankar VG (2001). Report BARC-2001/E/006: Earthquake sequence of 1991 from Valsad Region, Guajrat.

[17] Pulinets S, Boyarchuk K (2004). Ionospheric Precursors of Earthquakes.

[18] Liu JY, Chen CH, Chen YI, Yen HY, Hattori K, Yumoto K (2006). Seismo-geomagnetic anomalies and M5.0 earthquakes observed in Taiwan during 1988–2001. Phys. Chem. Earth.

[19] Nemec F, Santolík O, Parrot M, Berthelier JJ (2008). Spacecraft observations of electromagnetic perturbations connected with seismic activity. Geophys Res Lett.

[20] Rozhnoi AM, Solovieva OM, Schwingenschuh K, Boudjada M, Biagi PF, Maggipinto T, Castellana L, Ermini A, Hayakawa M (2009). Anomalies in VLF radio signals prior the Abruzzo earthquake (M = 6.3) on 6 April 2009. Nat. Hazards Earth Syst. Sci.

[21] Kasahara Y, Muto F, Horie T, Yoshida M, Hayakawa M, Ohta K, Rozhnoi A, Solovieva M, Molchanov OA (2008). On the statistical correlation between the ionospheric perturbations as detected by subionospheric VLF/LF propagation anomalies and earthquakes. Nat. Hazards Earth Syst. Sci.

[22] Hayakawa M (2004). Electromagnetic phenomena associated with earthquakes: A frontier in terrestrial electromagnetic noise environment. Recent Res. Dev. Geophys.

[23] Krueger AP, Reed EJ (1976). Biological impact of small air ions. Science.

[24] Logan JM (1977). Animal behavior and earthquake prediction. Nature.

[25] Kirschvink JL (2000). Earthquake prediction by animals: Evolution and sensory perception. Bull. Seismol. Soc. Am.

[26] Morton IL (1988). Headaches prior to earthquakes. Int. J. Biometeorol.

[27] Rose MS, Verhoef MJ, Ramcharan S (1995). The relationship between chinook conditions and women’s illness-related behaviours. Int. J. Biometeorol.

[28] Piorecky J, Becker WJ, Rose MS (1997). Effect of chinook winds on the probability of migraine headache occurrence. Headache.

[29] Balk M, Bose M, Ertem G, Rogoff DA, Rothschild LJ, Freund FT (2009). Oxidation of water to hydrogen peroxide at the rock-water interface due to stress-activated electric currents in rocks. Earth Planet. Sci. Lett.

[30] İnan ST, Akgül C, Seyis R, Saatçılar S, Baykut S, Ergintav S, Baş M (2008). Geochemical monitoring in the Marmara region (NW Turkey): A search for precursors of seismic activity. J Geophys Res.

[31] Pérez NM, Hernández-del-Valle G, Igarashi G, Trujillo I, Nakai S, Sumino H, Wakita H (2008). Searching and detecting earthquake geochemical precursors in CO_2_-rich groundwaters from Galicia, Spain. Geochem. J.

[32] Biagi PF, Piccolo R, Ermini A, Fujinawa Y, Kingsley SP, Khatkevich YM, Gordeev EI (2001). Hydrogeochemical precursors of strong earthquakes in Kamchatka: further analysis. Nat. Hazards Earth Syst. Sci.

[33] Balderer W, Leuenberger F (2002). Effects of the Cinarcik-Ismit August 17, 1999 earthquake on the composition of thermal and mineral waters as revealed by chemical and isotope investigations. Geophys. Int.

[34] Balderer W, Leuenberger F, Sen P, Das NK (2006). Observation of Fluorescence Spectra of Ground Water in Areas of Tectonic Activity: Could It Act as a Precursor?. Geochemical Precursors for Earthquakes.

[35] Michetti AM (2005). Active tectonics and seismic hazard in the Central Western Southern Alps: A review. Geophys. Rese. Abstr.

[36] Saran M, Summer KH (1999). Assaying for hydroxyl radicals: Hydroxylated terephthalate is a superior fluorescence marker than hydroxylated benzoate. Free Radic. Res.

[37] Singh RP, Kumar JS, Zlotnicki J, Kafatos M (2010). Satellite detection of carbon monoxide emission prior to the gujarat earthquake of 26 January 2001. Appl. Geochem.

[38] Milne J, Lee AW (1939). Earthquakes and Other Earth Movements.

[39] Turcotte DL (1991). Earthquake prediction. Annu. Rev. Earth Planet. Sci.

[40] Ikeya M, Furuta H, Kajiwara N, Anzai H (1996). Ground electric field effects on rats and sparrows: Seismic anomalous animal behaviors (SAABs). Jpn. J. Appl. Phys.

[41] Wang K, Chen QF, Sun S, Wang A (2006). Predicting the 1975 Haicheng Earthquake. Bull. Seismol. Soc. Am.

[42] Zhu F, Wu G (1982). Haicheng Earthquake.

[43] Grant RA, Halliday T (2010). Predicting the unpredictable: Evidence of pre-seismic anticipatory behaviour in the common toad. J. Zool.

[44] Gittins SP, Parker AG, Slater FM (1980). Population characteristics of the common toad (Bufo bufo) visiting a breeding site in mid-Wales. J. Anim. Ecol.

[45] Fidani C (2010). The earthquake lights (EQL) of the 6 April 2009 Aquila earthquake in Central Italy. Nat. Hazards Earth Syst. Sci.

[46] Serpieri A (1873). Nuove osservazioni sul terremoto avvenuto in Italia il 12 marzo 1873 e riflessioni sul presentimento degli animali per i terremoti. Rendiconti del R. Istituto lombardo.

[47] Reading CJ, Clarke RT (1983). Male breeding behaviour and mate acquisition in the common toad, Bufo bufo. J. Zool.

[48] Liu JY, Chuo YJ, Shan SJ, Tsai YB, Chen YI, Pulinets SA, Yu SB (2004). Pre-earthquake ionospheric anomalies. Ann. Geophys.

[49] Chen YI, Liu JY, Tsai YB, Chen CS (2004). Statistical test for pre-earthquake ionospheric anomaly. Terr. Atmo. Ocean. Sci.

[50] Hayakawa M, Kasahara Y, Nakamura T, Muto F, Horie T, MAekawa S, Hobara Y, Rozhnoi AA, Solivieva M, Molchanov OA (2010). A statistical study on the correlation between lower ionospheric perturbations as seen by subionospheric VLF/LF propagation and earthquakes. J. Geophys. Res.

[51] Hayakawa M, Kasahara Y, Hobara TNY, Rozhnoi A, Solovieva M, Molchanov OA (2010). On the correlation between ionospheric perturbations as detected by subionospheric VLF/LF signals and earthquakes as characterized by seismic intensity. J. Atmos. Sol.-Terr. Phys.

[52] Tsolis GS, Xenos TD (2010). A qualitative study of the seismo-ionospheric precursors prior to the 6 April 2009 earthquake in L’Aquila, Italy. Nat. Hazards Earth Syst. Sci.

[53] Eftaxias K, Balasis G, Contoyiannis Y, Papadimitriou C, Kalimeri M, Athanasopoulou L, Nikolopoulos S, Kopanas J, Antonopoulos G, Nomicos C (2010). Unfolding the procedure of characterizing recorded ultra low frequency, kHZ and MHz electromagetic anomalies prior to the L’Aquila earthquake as pre-seismic ones—Part II. Nat. Hazards Earth Syst. Sci.

[54] Eftaxias K, Athanasopoulou L, Balasis G, Kalimeri M, Nikolopoulos S, Contoyiannis Y, Kopanas J, Antonopoulos G, Nomicos C (2009). Unfolding the procedure of characterizing recorded ultra low frequency, kHZ and MHz electromagetic anomalies prior to the L’Aquila earthquake as pre-seismic ones—Part I. Nat. Hazards Earth Syst. Sci.

[55] Fidani C (2009). Electromagnetic Signals Recorded by Perugia and S. Procolo (Fermo) Stations before the Aquila Earthquakes.

[56] Derr JS, St-Laurent F, Freund FT, Thériault R, Gupta H (2010). Earthquake Lights. Encyclopedia of Solid Earth Geophysics.

[57] Derr JS (1973). Earthquake lights: A review of observations and present theories. Bull. Seismol. Soc. Am.

[58] Buskirk RE, Frohlich CL, Latham GV (1981). Unusual animal behavior before earthquakes: A review of possible sensory mechanisms. Rev. Geophys.

[59] Ikeya M (2004). Earthquakes and Animals: From Folk Legends to Science.

[60] Ikeya M, Yamanaka C, Mattsuda T, Sasaoka H, Ochiai H, Huang Q, Ohtani N, Komuranani T, Ohta M, Ohno Y, Nakagawa T (2000). Electromagnetic pulses generated by compression of granitic rocks and animal behavior. Episodes.

[61] Witze A (2009). The sleeping dragon. Nature.

[62] World Stress Map Project http://dc-app3-14.gfz-potsdam.de/.

[63] King BV, Freund F (1984). Surface charges and subsurface space charge distribution in magnesium oxide containing dissolved traces of water. Phys. Rev.

[64] Hayakawa M, Hattori K, Ohta K (2007). Monitoring of ULF (ultra-low-frequency) geomagnetic variations associated with earthquakes. Sensors.

[65] Molchanov OA, Schekotovm A, Federov E, Belyaev G, Gordeev EI (2003). Preseismic ULF electromagnetic effect from observation at Kamchatka. Nat. Hazards Earth Syst. Sci.

[66] Hayakawa M, Ito T, Hattori K, Yumoto K (2000). ULF electromagnetic precursors for an earthquake at Biak, Indonesia on February 17, 1996. Geophys. Res. Lett.

[67] Rycroft MJ, Harrison RG, Nicoll KA, Mareev EA (2008). An overview of earth’s global electric circuit and atmospheric conductivity. Space Sci. Rev.

[68] Zhao B, Wang M, Yu T, Guirong X, Wan W, Liu L (2010). Ionospheric total electron content variations prior to the 2008 Wenchuan Earthquake. Int. J. Remote Sens.

[69] Liu JY, Chen CH, Chen YI, Yang WH, Oyama KI, Kuo KW (2010). A statistical study of ionospheric earthquake precursors monitored by using equatorial ionization anomaly of GPS TEC in Taiwan during 2001–2007. J. Asian Earth Sci.

[70] Liu JY, Chuo Y, Shan S, Tsai Y, Chen Y, Pulinets S, Yu SB (2004). Pre-earthquake ionospheric anomalies registered by continuous GPS TEC measurements. Ann. Geophys.

[71] Livingstone DR (2003). Oxidative stress in aquatic organisms in relation to pollution and aquaculture. Revue Méd. Vét.

[72] Lushchack VI (2011). Environmentally induced oxidative stress in aquatic animals. Aquat. Toxicol.

[73] Fridovich I (1995). Superoxide radical and superoxide dismutases. Annu. Rev. Biochem.

[74] Davies KJ, Delsignore ME (1987). Protein damage and degradation by oxygen radicals. III. Modification of secondary and tertiary structure. J. Biol. Chem.

[75] Imlay JA, Linn S (1988). DNA damage and oxygen radical toxicity. Science.

[76] Michaelidis B, Ouzounis C, Paleras A, Pörtner HO (2005). Effects of long-term moderate hypercapnia on acid-base balance and growth rate in marine mussels Mytilus galloprovincialis. Mar. Ecol. Prog. Ser.

[77] Reid SD, Dockray JJ, Linton TK, McDonald DG, Wood CM (1997). Effects of chronic environmental acidification and a summer global warming scenario: Protein synthesis in juvenile rainbow trout (Oncorhynchus mykiss). Can. J. Fish. Aquat. Sci.

[78] Amitai Y, Zlotogorski Z, Golan-Katzav V, Wexler A, Gross D (1998). Neuropsychological impairment from acute low-level exposure to carbon monoxide. Arch. Neurol.

[79] Feder ME, Burggren WW (1992). Environmental Physiology of the Amphibians.

[80] Vitt LJ, Caldwell JP, Wilbur HM, Smith DC (1990). Amphibians as harbingers of decay. BioScience.

[81] Valko M, Morris H, Cronin MT (2005). Metal, toxicity and oxidative stress. Curr. Med. Chem.

[82] Mahapatra PK, Mohanty-Hejmadi P, Chainy GB (2001). Specific limb abnormalities induced by hydrogen peroxide in tadpoles of Indian jumping frog, *Polypedates maculatus*. Indian J. Exp. Biol.

[83] Sadinski WJ, Dunson WA (1992). A multilevel study of effects of low pH on amphibians of temporary ponds. J. Herpetol.

[84] Alvarado RH, Cox TC (1985). Action of polyvalent cations on sodium transport across skin of larval and adult Rana catesbeiana. J. Exp. Zool.

[85] Dobrovolsky IP, Zubkov SI, Miachkin VI (1979). Estimation of the size of earthquake preparation zones. Pure Appl. Geophys.

